# Supplementary material for: Coriander (Coriandrum sativum) Cultivation Combined with Arbuscular Mycorrhizal Fungi Inoculation and Steel Slag Application Influences Trace Elements-Polluted Soil Bacterial Functioning
Source: Plants (Basel). 2023 Jan 31;12(3):618. doi: 10.3390/plants12030618 (PMC9920375; doi:10.3390/plants12030618)
Supplement: Supplementary file 1 [file plants-12-00618-s001.zip › plants-2159016-supplementary.pdf]

Table S1. Experimental design of plots describing all 20 modalities studied

Modality				Concentration (t/ha)
**Non-inoculated**	Unvegetated	*Unamended*		
		Ladle slag		*1.5*
				45
		Oven slag		*1.5*
				45
	Vegetated	*Unamended*		
		Ladle slag		*1.5*
				45
		Oven slag		*1.5*
				45
**Inoculated**	Unvegetated	*Unamended*		
		Ladle slag		*1.5*
				45
		Oven slag		*1.5*
				45
	Vegetated	*Unamended*		
		Ladle slag		*1.5*
				45
		Oven slag		*1.5*
				45

Table S2. Influence of amendments and vegetation (V: vegetated, UV: unvegetated, NI: not-inoculated, I: inoculated, LS: ladle slag and OS: oven slag, NA: not-amended, 1.5 and 45 $t.ha^{-1}$) on most abundant phyla relative abundance. One way ANOVA ± standard error. Means followed by the same lowercase letter are not significantly different, by one-way ANOVA comparison and post-hoc Fisher test (α = 0.05).

**Phyla**	**V**	**UV**	**NI**	**I**	**LS**	**OS**	**NA**	**1.5t.ha^{-1}**	**45t.ha^{-1}**
Actinobacteria	**0.62±0.013a**	**0.50±0.013b**	0.56±0.02a	0.57±0.02a	0.58±0.019a	0.53±0.019a	0.58±0.027a	0.58±0.019a	0.52±0.019a
Proteobacteria	**0.18±0.004b**	**0.20±0.004a**	0.19±0.005a	0.19±0.005a	**0.20±0.005a**	**0.19±0.005b**	**0.17±0.007b**	**0.19±0.005ab**	**0.20±0.005a**
Gemmatimonadota	**0.06±0.007b**	**0.14±0.007a**	0.10±0.010a	0.10±0.010a	0.09±0.011a	0.11±0.011a	0.10±0.016a	0.09±0.011a	0.12±0.011a
Myxococcota	0.046±0.001a	0.045±0.001a	0.044±0.001a	0.047±0.001a	0.044±0.002a	0.046±0.002a	0.048±0.002a	0.62±0.01a	0.62±0.01a
Acidobacteriota	**0.018±0.002b**	**0.32±0.002a**	0.025±0.01a	0.024±0.01a	**0.022±0.002b**	**0.029±0.002a**	0.022±0.003a	0.024±0.002a	0.027±0.002a
Bacteroidota	**0.016±0.001b**	**0.021±0.001a**	0.019±0.001a	0.018±0.001a	**0.017±0.001b**	**0.020±0.001a**	0.017±0.001a	0.017±0.001a	0.020±0.001a
Thermoplasmatota	0.017±0.001a	0.016±0.001a	0.017±0.001a	0.017±0.001a	**0.019±0.001a**	**0.014±0.001b**	**0.018±0.002ab**	**0.014±0.001b**	**0.019±0.001a**
Patescibacteria	0.008±0.001a	0.009±0.001a	0.008±0.001a	0.009±0.001a	**0.009±0.001a**	**0.007±0.001b**	**0.009±0.001a**	**0.007±0.001b**	**0.009±0.001a**
Firmicutes	**0.007±0.001b**	**0.009±0.001a**	0.008±0.001a	0.008±0.001a	0.008±0.001a	0.008±0.001a	**0.009±0.001a**	**0.007±0.001b**	**0.009±0.001a**
Verrucomicrobiota	**0.004±0.001b**	**0.006±0.001a**	0.006±0.001a	0.005±0.001a	0.005±0.001a	0.006±0.001a	**0.004±0.001ab**	**0.004±0.001b**	**0.006±0.001a**
Nitrospirota	**0.004±0.000b**	**0.006±0.000a**	0.005±0.000a	0.005±0.000a	0.004±0.000a	0.005±0.000a	0.005±0.001a	0.005±0.000a	0.005±0.000a
Spirochaetota	0.004±0.000a	0.004±0.000a	0.004±0.000a	0.004±0.000a	0.004±0.000a	0.004±0.000a	0.004±0.001a	0.003±0.000a	0.005±0.000a
Campylobacterota	0.003±0.000a	0.003±0.000a	0.003±0.000a	0.003±0.000a	0.003±0.000a	0.002±0.000a	0.003±0.000a	0.003±0.000a	0.002±0.000a
Planctomycetota	**0.001±0.000b**	**0.002±0.000a**	0.002±0.000a	0.002±0.000a	0.002±0.000a	0.002±0.000a	0.001±0.000a	0.002±0.000a	0.002±0.000a
**Others phyla**	**0.007±0.000b**	**0.011±0.000a**	0.009±0.000a	0.008±0.000a	0.009±0.001a	0.009±0.001a	0.008±0.001a	0.009±0.001a	0.009±0.001a

Table S3. Influence of amendments and vegetation (V: vegetated, UV: unvegetated, NI: not-inoculated, I: inoculated, LS: ladle slag and OS: oven slag, NA: not-amended, 1.5 and 45 t.ha-1) on biolog substrats familly.

State	Amendment	Dose ($t.ha^{-1}$)	Inoculation	Carbohydrates	Amino acids	Carboxylic acetic acids	Amines amides	Polymers
Unvegetated	Unamended		NI	998,±254	658 ±185	908±109	124±40	420±45
			I	1119±231	735±120	924±136	165±38	434±61
	Ladle slag	1.5	NI	1092±665	607±102	889±220	128±40	428±108
			I	1382±377	883±103	1053±215	207±89	519±39
		45	NI	1006±268	741±66	885±135	138±47	500±87
			I	904±453	715±130	892±100	158±51	426±77
	Oven slag	1.5	NI	929±233	754±131	1041±239	181±34	425±69
			I	727±43	751±210	936±219	235±130	412±53
		45	NI	718±447	650±54	821±177	172±52	364±43
			I	879±348	661±118	914±144	123±60	421±70
Vegetated	Unamended		NI	1470±192	865±78	1105±48	213±28	528±94
			I	1710±311	876±115	1245±101	207±53	553±129
	Ladle slag	1.5	NI	1685±250	736±22	1230±159	212±38	523±86
			I	1612±302	785±163	1081±270	193±32	567±127
		45	NI	1371±459	823±112	1113±233	225±25	460±85
			I	1283±397	830±86	961±104	179±53	529±81
	Oven slag	1.5	NI	1579±316	792±132	1065±146	192±83	575±101
			I	1479±219	767±116	1153±135	226±42	505±113
		45	NI	1851±137	690±99	1232±54	182±32	620±126
			I	1685±407	835±83	1237±142	195±47	546±109
